# Supplementary material for: A Comparison of Lymphoid and Myeloid Cells Derived from Human Hematopoietic Stem Cells Xenografted into NOD-Derived Mouse Strains
Source: Microorganisms. 2023 Jun 10;11(6):1548. doi: 10.3390/microorganisms11061548 (PMC10300940; doi:10.3390/microorganisms11061548)
Supplement: Supplementary file 1 [file microorganisms-11-01548-s001.zip › microorganisms-2445585-supplementary.pdf]

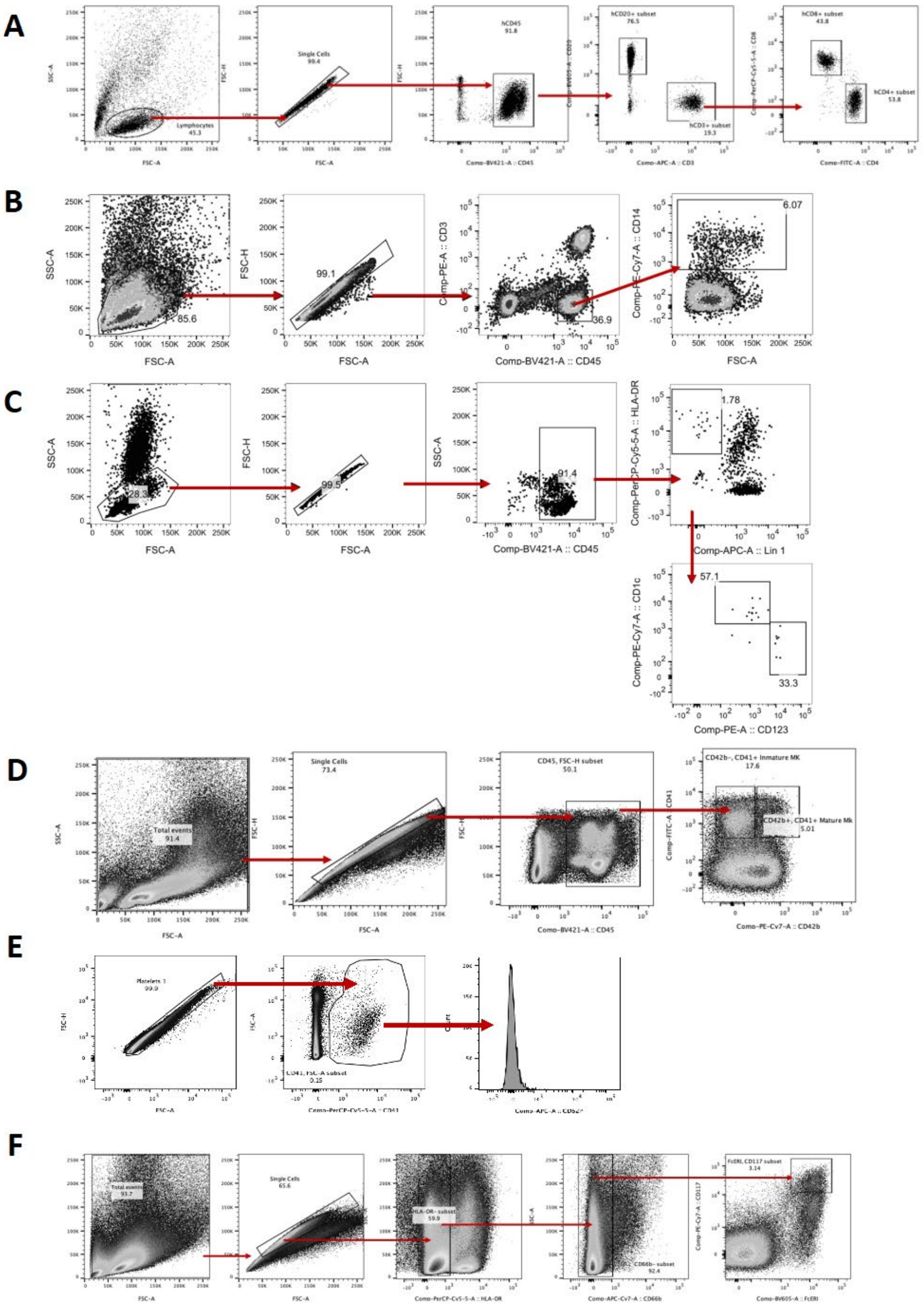

Supplemental Figure S1. Representative gating strategy from blood or tissue cells for the identification of individual cell populations in hu-NSG, hu-NCG, hu-SGM3 and hu-EXL. (A) human lymphocyte T and B, (B) human monocytes, (C) human dendritic cells, (D) human megakaryocytes, (E) human platelets, (F) human mast cells.
